# Effects of Defatted and Hydrolyzed Black Soldier Fly Larvae Meal as an Alternative Fish Meal in Weaning Pigs

**DOI:** 10.3390/ani14111692

**Published:** 2024-06-05

**Authors:** Jihwan Lee, Younguk Park, Dongcheol Song, Seyeon Chang, Jinho Cho

**Affiliations:** 1 Swine Science Division, National Institute of Animal Science, Rural Development Administration, Cheonan 31000, Republic of Korea; junenet123@naver.com; 2 Chungcheongbuk-do Agricultural Research and Extension Service, Cheongju 28130, Republic of Korea; papark1@korea.kr; 3 Department of Animal Science, Chungbuk National University, Cheongju 28644, Republic of Korea; paul741@daum.net (D.S.); angella2425@naver.com (S.C.)

**Keywords:** black soldier fly larvae meal, growth performance, nutrient digestibility, weaning pigs

## Abstract

**Simple Summary:**

Substitution with black soldier fly larvae meal (BLM, *Hermetia illucens* L.) has been studied as a possible means of replacing expensive protein sources such as soybean meal and fish meal. However, availability of BLM can differ depending on the substrate and its processing. We investigated the effect of supplementation with defatted and hydrolyzed BLM as an alternative to fish meal in weaning pigs. We found that supplementation with defatted BLM improved nutrient digestibility, growth performance, and economic returns when compared with fish meal (FM) in weaning pigs.

**Abstract:**

In Experiment 1, a total of eighteen crossbred ([Landrace × Yorkshire] × Duroc) barrows with an initial body weight of 6.74 ± 0.68 kg were randomly divided into three dietary treatments (one pig per cage and six replicates per treatment) and housed in metabolic cages that were equipped with a feeder and slatted floor to collect urine and feces. In Experiment 2, a total of 96 crossbred ([Landrace × Yorkshire] × Duroc) barrows with an initial body weight of 8.25 ± 0.42 kg were used in the 6-week trial. The pigs were randomly divided into three dietary treatments (three pigs per pen and eight replicates per treatment). In Experiment 1, nutrient composition of defatted black soldier fly larvae meal (BLM) was superior to that of hydrolyzed BLM but lower than that of fish meal (FM). Also, defatted BLM and FM had better apparent total track digestibility (ATTD) of crude protein (CP) and better nitrogen retention (*p* < 0.05) than hydrolyzed BLM, but there was no significant difference (*p* > 0.05) between defatted BLM and FM. In Experiment 2, defatted BLM improved (*p* < 0.05) average daily gain (ADG), feed conversion ratio (FCR), and feed cost per kg gain (FCG) compared with FM. Defatted BLM could replace soybean meal and fish meal as an alternative protein source for weaning pigs.

## 1. Introduction

As global population and incomes increase, the consumption of meat originating from monogastric animals is growing, and among these animals, approximately one-third of global meat production is from pork [1,2,3]. Simultaneously, this globally increased consumption has caused high pressure on feed resources [4]. On average, 33% of arable land is used to produce livestock feed, and the livestock produced accounts for 25% of all human protein production [5]. It is difficult to produce feed protein for livestock due to limited arable land and unstable fish catches [6]. Protein feed ingredients are one of the most expensive types of feed (accounting for approximately 60% to 80% of the total feed cost) and limiting feed ingredients in livestock diets [7]. Thus, extensive studies have been conducted to find suitable feed protein alternatives that do not alter the growth performance in animals. Insect meal is known as having high quality protein as well as other essential nutrients for livestock, such as fat and minerals, and even active compounds such as antibiotics that can have a positive effect on livestock health [8,9]. Moreover, insects can consume animal manure and food waste, which reduces environmental pollution and converts the waste into protein [10,11]. In particular, black soldier fly larvae meal (BLM; *Hermetia illucens*) is easily obtained since these insects are widespread in the tropics and warm temperate regions of the world [12]. The meal contains 7–39% fat and 37–63% protein on a dry matter basis, and has an essential amino acid content similar to that of fish meals [8]. Therefore, BLM has been considered as an effective alternative to fish meals, and many researchers have conducted investigations to evaluate the effects of BLM supplementation on growth performance, nutrient digestibility, blood profiles, and even gut health in pigs in various phases [13,14,15,16].

However, the quality and/or composition of insect meals can be different depending on the substrate used for their rearing. It is also dependent on feed processing, which can be used to increase availability and reduce anti-nutritive factors [17,18]. Cho et al. [17] reported that hydrolyzed mealworm larvae meal improved in vitro digestibility and even apparent ileal digestibility (AID) of nutrients compared with defatted mealworm larvae meal. Moreover, the processing and thermal-conditioning techniques showed a tendency to improve digestibility of amino acids (AAs) in BLM [19]. However, although protein ingredients are crucial during the weaning period, which is the most important phase for early growth, there is little research comparing hydrolyzed and defatted black soldier fly larvae, which are protein sources. Therefore, we conducted an investigation to compare the effect of hydrolyzed and defatted BLM on growth performance, nutrient digestibility, economic returns, and blood profiles in pigs.

## 2. Materials and Methods

### 2.1. Ethics

The protocol for this study was approved by the Institutional Animal Care and Use Committee of Chungbuk National University, Cheongju, Republic of Korea (No. CBNUA-2184-23-02).

### 2.2. Black Soldier Fly Larvae Meal Sample

Defatted BLM obtained from Agricultural Research and Extension Services (Cheongju, Republic of Korea) and hydrolyzed BLM obtained from Jeju National University (Jeju, Republic of Korea) were evaluated (Table 1). The defatted BLM used black soldier flies harvested by rearing 3rd-instar larvae hatched from eggs on wet feed (food waste; 70% moisture) for 10 days. After first drying the harvested black soldier fly larvae using a microwave dryer (M-200, Entomo, Siheung, Republic of Korea), secondary drying was performed using a roaster (M-201, Entomo, Siheung, Republic of Korea) to remove moisture to within 1%. After drying, the larvae were milked using a screw-type insect oil press machine (M-202, Entomo, Siheung, Republic of Korea), and then ground to 100 mesh or less using a fine grinder (M-205, Entomo, Siheung, Republic of Korea). The hydrolyzed BLM used black soldier flies harvested by rearing 3rd instar larvae for 10 days on 1:1 wet feed mixed with citrus peel and poultry offal. After harvesting, primary and secondary drying was carried out as for the defatted BLM and then hydrolyzed using alcalase at 50 °C for 12 h. After the reaction, it went through a concentration step and was dried with hot air. Afterward, it was ground to 100 mesh or less and powdered.

### 2.3. Digestibility Trial (Experiment 1)

#### 2.3.1. Experimental Animals and Design

A total of 18 crossbred ([Landrace × Yorkshire] × Duroc) barrows with initial body weight of 6.74 ± 0.68 kg at 4 weeks of age were randomly divided into three dietary treatments (1 pig per cage and 6 replicates per treatment) and housed in metabolic cages (45 × 55 × 45 cm) that were equipped with a feeder and slatted floors to collect urine and feces. This experiment was conducted in a room with a heater and fan installed to maintain a 23 ± 1.5 °C temperature with 83 ± 2.3% relative humidity and 0.25 ± 0.03 m/s wind speed.

#### 2.3.2. Diets and Feeding

The dietary treatments were as follows: (1) basal diet based on 5% fish meal (FM), (2) 100% fish meal replacement with defatted BLM, and (3) 100% fish meal replacement with hydrolyzed BLM. All diets were formulated to meet and exceed the National Research Council nutrient requirements for pigs (Table 2) [20]. The daily feeding allowance was calculated at 4% of mean BW of pigs (approximate 3.2 times the estimated metabolize energy (ME) requirement for maintenance ([197 kcal ME per kg body weight^0.60^]) in each period, divided into two equal portions and fed twice daily at 0800 and 1700 h. Pigs had free access to water throughout the experiment.

#### 2.3.3. Sampling and Analysis

Pigs were weighed at the beginning of experiment and the amount of feed consumed was recorded daily. The total experiment period was 18 days. The initial 5 days were considered the adaptation period to the diet followed by 4 days of total fecal and urine collection according to the marker to marker procedure for the first week [21]. This procedure was repeated in the second week. Briefly, feces were collected from when the first marker appeared in the feces after 6 days and collection stopped when the second marker appeared in the feces after 8 days. Urine was collected from the morning of the fourth day to the morning of the sixth day. Urine was collected into the buckets containing 50 mL of 6 mol/L H_2_SO_4_ which were located under the metabolism cages. During the collection period, feces and 20% of the urine were weighted and stored at −20 °C. Leftover feeds were collected and weighed daily to determine feed intake. All collected samples were dried at 65 °C for 72 h in a forced-air oven and ground through 1 mm screen and thoroughly mixed before further analysis. Dried samples, including feces, urine, and feeds, were analyzed in duplicate for dry matter (DM), gross energy (GE), crude protein (CP), and AAs, referring to AOAC methods [22]. The GE, nitrogen, and AAs were analyzed using a bomb calorimeter (Model 6400, Parr Instruments, Moline, IL, USA), a Kjeltec TM 8400 (FOSS Inc., Eden Prairie, MN, USA) with subsequent calculation of CP by a conversion factor of 6.25 for FM and a conversion factor of 4.67 for BLMs [23], and chromatography (Shimadzu model LC-10AT, Shimadzu, Kyoto, Japan), respectively. Using the analyzed values of nutrient concentration in feeds, the nutrient digestibility was calculated using the following equation: apparent total track digestibility (ATTD, %) = [(Ni − Nf) / Ni] × 100 where Ni is the total intake of energy of nutrient and Nf is the fecal output of energy or nutrient originating from the feeds. Nitrogen retention was calculated using following equation: nitrogen retention (%) = {Ni − ((Nf + Nu)/Ni)} × 100 where Ni is nitrogen (N) intake, and Nf and Nu are N output in feces and urine. For blood profiles, at the end of the week, blood was collected from the jugular vein of all pigs using 5 mL syringes and stored in a K_3_EDTA vacuum tube and a serum separator tube. Piglets had their feed and water withdrawn 12 h before blood collection. Samples were centrifuged at 12,500× *g* for 15 min at 4 °C. White blood cells (WBCs), red blood cells (RBCs), lymphocytes, and neutrophils; and blood urea nitrogen (BUN) and total protein concentrations were measured using an automatic blood analyzer (ADVIA 120, Bayer, Leverkusen, Germany) and automatic biochemistry blood analyzer (HITACHI 747; Hitachi, Tokyo, Japan), respectively.

### 2.4. Performance Trial (Experiment 2)

#### 2.4.1. Experimental Animals and Design

A total of 96 crossbred ([Landrace × Yorkshire] × Duroc) barrows at 4 weeks of age (average body weight of 8.25 ± 0.42 kg) were used in the 6-week trial. Pigs were randomly divided into three dietary treatments in a completely randomized block design based on initial BW. There were 3 pigs in a pen with 8 replicate pens for each treatment. The dietary treatments were same as those for Experiment 1: (1) basal diets based on 5% fish meal (FM), (2) 100% fish meal replacement with defatted BLM, and (3) 100% fish meal replacement with hydrolyzed BLM. All diets were formulated to meet and exceed the National Research Council nutrient requirements for pigs (Table 2) [20]. Each pen was equipped with a self-feeder and nipple drinker to allow ad libitum access to feed and water. This experiment was conducted in a room with a heater and fan installed to maintain at 23 ± 1.5 °C temperature with 83 ± 2.3% relative humidity and 0.25 ± 0.03 m/s wind speed.

#### 2.4.2. Sampling and Analysis

To measure growth performance, all piglets were weighed at the start of the experiment, after 2 weeks, after 4 weeks, and at the end of experiment (6 weeks), and feed consumption was recorded to calculate average daily gain (ADG), average daily feed intake (ADFI), and feed conversion ratio (FCR). Economic evaluation was based on the data obtained from each treatment. It was categorized as total weight gain and total feed intake. Total weight gain (TWG) and total feed intake (TFI) were calculated by multiplying the average daily gain and average daily feed intake by the total experimental period, respectively. Feed cost per weight gain (FCG) was calculated by multiplying the TFI by feed cost (USD 0.76/kg, Korea Institute for Animal Products Quality Evaluation, Sejong, Republic of Korea) and then dividing by TWG. When FCG was estimated, only feed cost was considered without taking into account other things such as electricity and porterage, because other expenses were charged, in common, to each treatment.

### 2.5. Statistical Analysis

All data were statistically analyzed by one-way analysis of variance (ANOVA) using JMP 16.0 (SAS Institute Inc., Cary, NC, USA) with post hoc analysis using Tukey’s honest significant difference (HSD) test. *p* < 0.05 was considered a statistically significant difference.

## 3. Results

### 3.1. Experimant 1

#### 3.1.1. Nutrient Digestibility

Effects of defatted and hydrolyzed BLM on ATTD of DM, CP, and GE as a fish-meal substitute in weaning pigs are presented in Table 3. There was no significant effect (*p* > 0.05) on ATTD of nutrients in week 1 among treatments. In week 2, there was no significant difference in ATTD (*p* > 0.05) of CP between FM and defatted BLM, while hydrolyzed BLM had lower ATTD of CP (*p* < 0.05) than other treatments. Although there was no significant difference (*p* > 0.05) in ATTD of DM and GE between treatments, FM and defatted BLM numerically increased ATTD of DM and GE compared with hydrolyzed BLM. The effects of defatted and hydrolyzed BLM as a fish-meal substitute on ATTD of amino acids in weaning pigs are presented in Table 4 and Table 5. There was no significant difference (*p* > 0.05) on ATTD of essential and non-essential amino acids between treatments in weeks 1 and 2. FM and defatted BLM numerically increased the ATTD of essential and non-essential amino acids in weeks 1 and 2 compared with hydrolyzed BLM.

#### 3.1.2. Nitrogen Retention

The effects of defatted and hydrolyzed BLM as a fish-meal substitute on N retention in weaning pigs are presented in Table 6. There was no significant difference (*p* > 0.05) on nitrogen retention in week 1 between treatments. Defatted BLM did not change (*p* > 0.05) nitrogen retention in week 2 compared to FM, while hydrolyzed BLM had lower nitrogen retention (*p* < 0.05) than FM.

#### 3.1.3. Blood Profiles

The effects of defatted and hydrolyzed BLM as a fish-meal substitute on blood profiles in weaning pigs are presented in Table 7. There was no significant difference (*p* > 0.05) on WBCs, RBCs, lymphocytes, neutrophils, or total protein concentration between treatments. Defatted BLM did not change (*p* > 0.05) BUN concentration compared to FM, while hydrolyzed BLM had higher BUN concentration (*p* < 0.05) than FM.

### 3.2. Experimant 2

#### Production Performance

The effects of defatted and hydrolyzed BLM as a fish-meal substitute on production performance in weaning pigs are presented in Table 8. There was no significant difference (*p* > 0.05) in BW between treatments for the entire experiment except for week 6. After 6 weeks, pigs fed defatted BLM had a higher BW (*p* < 0.05) than those fed FM. Neither type of BLM affected (*p* > 0.05) ADG, ADFI, or FCR during weeks 0 to 2 weeks and weeks 4 to 6 compared to FM. Defatted BLM significantly increased (*p* < 0.05) ADG during weeks 2 to 4 compared with FM. During the entire experiment, the defatted BLM group had a higher ADG and FCR (*p* < 0.05) than the FM group. However, there was no significant difference (*p* > 0.05) in ADG during weeks 2 to 4, or in ADG and FCR during weeks 0 to 6 weeks between pigs fed defatted or hydrolyzed BLM.

There was no significant difference (*p* > 0.05) in FCG during weeks 0 to 2 weeks and weeks 4 to 6 weeks between treatments. During weeks 2 to 4 and 0 to 6, defatted BLM significantly increased (*p* < 0.05) FCG compared with FM although there was no significant difference (*p* > 0.05) between the two types of BLM.

## 4. Discussion

According to previous studies, CP concentration of BLM ranged from 30 to 51% although their substrates were different [14,24]. Furthermore, recent studies have reported that full fat and defatted BLM had 41% and 71.2% CP concentration, respectively [25,26]. In our study, CP concentration of defatted and hydrolyzed BLM represented 56.02% and 59.97%, respectively, although their CP concentrations were lower than those of conventional FM. Likewise, defatted BLM had better amino acid profiles of both essential and non-essential amino acids than hydrolyzed BLM despite the fact that their components did not reach those of FM. Lys, classified as belonging to the epsilon amino group, is most susceptible to heat damage and thus Lys concentration of heat-damaged feed ingredients could be decreased while heat damage usually does not affect CP concentration [27]. The Lys:CP ratio has been considered as an indicator of heat damage [28,29]. In this study, the Lys:CP ratio in FM was 0.083, whereas the ratios in defatted and hydrolyzed BLM were 0.062 and 0.057, respectively. High pressure is required for the defatting process, which probably results in heat damage to BLM, leading to a slight reduction in the Lys:CP ratio compared with that of FM. Moreover, the hydrolyzing process is more necessary than the defatting process, and hydrolyzed BLM, therefore, had the lowest Lys concentration compared with other ingredients. According to Dabbou et al. [30], BLM contains a greater amount of lipids, which could represent a higher energy concentration. We found that hydrolyzed BLM had a slightly higher GE concentration than FM and defatted BLM. This finding may be due to the high EE concentration of hydrolyzed BLM. The EE concentration of BLM ranges from 3.4% to 38.6% [31]. Previous studies have reported that nutrient components including CP and EE concentration of insect meals could be altered depending on species, growth phase, harvest point, and processing technique [8,32,33].

In the digestibility trial, no significant difference was observed between treatments for ATTD of DM, CP, or GE in week 1, and ATTD of DM and GE in week 2. Likewise, there was no significant difference in ATTD of indispensable and dispensable amino acids between treatments. Nevertheless, defatted BLM and FM increased ATTD of CP in week 2 compared with hydrolyzed BLM, while there was no significant difference between FM and defatted BLM. Our results were in agreement with those of previous studies which found that dietary BLM supplementation and even partial replacement of soybean meal with defatted BLM did not change nutrient digestibility compared to a basal diet in piglets [16,34]. The main factor of BLM, which could affect nutrient digestibility of livestock, is chitin concentration. BLM is known to contain chitin and this even has a negative effect on nutrient digestion, especially protein and lipid in the animals [35]. Some in vivo studies have reported that chitin supplementation can decrease CP and OM digestibility compared with control diets [36,37]. Likewise, an in vitro study showed that chitin concentration in BLM was negatively correlated with DM, OM, and CP digestibility [38]. Although we did not estimate chitin concentration of both BLMs in this study, it is considered that the lowest ATTD of CP in hydrolyzed BLM may be due to the chitin concentration which is included in the BLM. Our results for N retention and BUN could indirectly prove the above-mentioned mechanism. Chitin, one of the major component of the insect cuticle, increases the actual protein content due to chitinous nitrogen [39,40]. Compared with defatted BLM and FM, N excretion in feces in hydrolyzed BLM was increased at each time point and thus, hydrolyzed BLM had a lower N retention percentage than the other treatments, especially during week 2. Moreover, hydrolyzed BLM had higher BUN concentration, which is inversely correlated to protein utilization in blood, than FM and defatted BLM. As a consequence, increased N excretion of hydrolyzed BLM in feces may mainly be due to the chitin concentration, causing poor N digestibility of hydrolyzed BLM. On the other hand, ATTD of CP and BUN concentrations in blood in pigs fed defatted BLM was similar to that of FM. Rumpold and Schlüter [41] suggested the partial removal of chitin through high pressure processing as a method to solve the reduction in digestion of BLM. Therefore, the positive effects of defatted BLM on nutrient digestibility may have been mainly due to reduction in chitin through the use of high pressure in the defatting process and the preservation of Lys concentration compared with hydrolyzed BLM. However, some studies have shown that DM and CP digestibility was increased by gradually increasing dried mealworm in diets for piglets [42]. Also, according to our previous study, defatted BLM had similar GE and CP digestibility to that of FM, while hydrolyzed BLM had higher CP digestibility than defatted BLM in broilers [43]. These inconsistent results may be mainly due to differences in animals, components of BLM, and the environment.

In the performance trial, defatted BLM supplementation during the entire experimental period improved BW, ADG, and FCR compared with FM. This result was in agreement with a previous study which reported that mealworm supplementation improved growth performance, although the type of insect used and the affected phase were different [42,44]. Unlike our results, other studies have reported that heterogeneous insect meals including full fat and defat types did not influence growth performance and 3.5% FM replacement with defatted BLM also did not affect growth performance of pigs [14,16,34,45,46]. Also, 75% soybean meal replacement with BLM slightly reduced growth performance including growth, feed intake, and protein efficiency in piglets [15]. These inconsistent results may be due to the nutrition difference depending on species and substrate for rearing the insects. As mentioned above, the CP concentration ranged from about 36% to 48%, and EE concentration ranged from about 37% to 48%, although the same dried full-fat BLM was used [16,47,48]. Furthermore, the contrasting results in studies related to the effect of partial BLM supplementation on growth performance could be attributed to the chitin concentration in insect meal. The existence of chitin brings positives and negatives. For example, as mentioned above, chitin could inhibit the nutrient digestibility of pigs. However, many studies have reported that increasing chitin through increased BLM supplementation could activate or boost the innate immune responses in animals and improve intestinal immunoglobulin A (IgA) [6,49,50]. Therefore, the improvement in growth in this study could be attributed to the positive effects on immunity of the chitin contained in both BLMs. In the economic evaluation in this study, defatted BLM and hydrolyzed BLM as FM replacement slightly decreased FCG compared with FM. This result may be attributed to increased ADG and BW in both BLM groups with no alteration in FI. Moreover, the price of defatted and hydrolyzed BLM was USD 2.88/kg and USD 2.95/kg, respectively, and they were cheaper than FM (USD 3.18/kg). Additionally, this price advantage may be attributed to improved economic evaluations of both BLMs.

## 5. Conclusions

This study suggests that full replacement of fish meal with defatted black soldier fly larvae meal (BLM) did not have a negative effect in terms of nutrient digestibility, but rather, had a positive effect in terms of growth performance and economic evaluation in piglets in Experiments 1 and 2. Therefore, defatted BLM can replace fish meal as an alternative protein source for weaning pigs. However, since the nutrient composition could be different depending on growth phase, substrate, and processing technique, further studies should be performed while maintaining stable nutrient components from BLM.

## Figures and Tables

**Table 1:** Chemical composition of fish meal, defatted black soldier fly larvae (BLM), and hydrolyzed black soldier fly larvae meal (BLM) ^1^.

Items	FM	Defatted BLM	Hydrolyzed BLM
General Components			
GE, kcal/kg	4542.20	4578.12	4638.92
DM, %	93.00	93.25	93.41
CP, %	67.00	56.02	59.97
EE, %	9.00	6.26	11.44
CF, %	0.30	10.28	8.62
Ash, %	10.02	16.93	10.02
Essential Amino Acids, %			
Lys	5.60	3.51	3.40
Thr	3.10	2.20	2.00
Trp	1.05	0.61	0.54
Met	2.56	1.03	0.83
Phe	2.22	2.27	2.42
Ile	2.71	2.29	2.41
Leu	4.42	3.80	3.87
His	2.21	1.70	1.48
Arg	4.37	2.77	2.68
Val	3.48	3.85	3.55
Non-Essential Amino Acids, %			
Ala	4.73	3.55	4.24
Asp	6.50	5.03	5.11
Glu	7.85	6.11	6.14
Gly	4.70	3.08	3.03
Ser	2.77	2.38	2.07
Tyr	1.83	3.28	3.66
Cys	0.91	0.55	0.37
Pro	2.83	3.27	3.55

^1^ Abbreviations: BLM, black soldier fly larvae; CP, crude protein; EE, ether extract; CF, crude fiber; Lys, lysine; Thr, threonine; Met, methionine; Phe, phenylalanine; Ile, isoleucine; Leu, leucine; His, histidine; Arg, arginine; Val, valine; Ala, alanine; Asp, aspartic acid; Gly, glycine; Ser; serine; Tyr, tyrosine; Cys, cysteine; Pro, proline.

**Table 2:** Chemical composition of fish meal (FM), defatted black soldier fly larvae (BLM), and hydrolyzed black soldier fly larvae meal (BLM) (Exp. 1 and 2) ^1^.

Items	FM	Defatted BLM	Hydrolyzed BLM
Ingredients, %			
Corn	36.43	36.12	36.07
Extruded corn	15.00	15.00	15.00
Lactose	10.00	10.00	10.00
Soybean meal, 44% CP	14.50	14.81	14.86
Soy protein concentrate, 65% CP	8.00	8.00	8.00
Fishmeal	5.00	-	-
Defatted BLM	-	5.00	-
Hydrolyzed BLM	-	-	5.00
Whey	5.00	5.00	5.00
Soy oil	2.20	2.20	2.20
Monocalcium phosphate	1.26	1.26	1.26
Limestone	1.40	1.40	1.40
L-Lysine-HCl, 78%	0.06	0.06	0.06
DL-Methionine, 50%	0.15	0.15	0.15
Choline chloride, 25%	0.10	0.10	0.10
Vitamin premix ^2^	0.25	0.25	0.25
Trace mineral premix ^3^	0.25	0.25	0.25
Salt	0.40	0.40	0.40
Total	100.00	100.00	100.00
Calculated Value			
ME, kcal/kg	3493	3493	3493
CP, %	20.55	20.55	20.55
Lysine, %	1.55	1.55	1.55
Methionine, %	0.41	0.41	0.41
Analyzed Value			
ME, kcal/kg	3442.02	3426.98	3427.33
CP, %	19.61	19.56	19.60
Lysine, %	1.48	1.44	1.43
Methionine, %	0.49	0.43	0.39

^1^ Abbreviation: FM, basal diet with fishmeal; defatted BLM, basal diet with 100% replacement of fishmeal with defatted BLM; hydrolyzed BLM, basal diet with 100% replacement of fishmeal with hydrolyzed BLM; CP, crude protein; ME, metabolize energy. ^2^ Provided per kg of complete diet: vitamin A, 11,025 IU; vitamin D_3_, 1103 IU; vitamin E, 44 IU; vitamin K_3_, 4.4 mg; riboflavin, 8.3 mg; niacin, 50 mg; thiamine, 4 mg; d-pantothenic, 29 mg; choline, 166 mg; and vitamin B_12_, 33 mg. ^3^ Provided per kg of complete diet without zinc: Cu (as CuSO_4_·5H_2_O), 12 mg; Mn (as MnO_2_), 8 mg; I (as KI), 0.28 mg; and Se (as Na_2_SeO_3_·5H_2_O), 0.15 mg.

**Table 3:** Effects of defatted and hydrolyzed black soldier fly larvae meal (BLM) as a fish-meal substitute on apparent total tract digestibility (ATTD) of DM, CP, and GE in weaned pigs (Exp. 1) ^1^.

Items, %	FM	Defatted BLM	Hydrolyzed BLM	SE	*p*-Value
Week 1					
DM	84.55	85.40	84.03	1.806	0.865
CP	74.98	76.04	75.07	2.842	0.958
GE	83.80	84.95	82.29	1.913	0.624
Week 2					
DM	84.50	85.42	82.09	1.368	0.240
CP	80.27 ^a^	80.36 ^a^	73.19 ^b^	1.903	0.026
GE	84.38	84.96	80.66	1.489	0.120

^1^ Abbreviation: DM, dry matter; CP, crude protein; GE, gross energy; FM, basal diet with fishmeal; defatted BLM, basal diet with 100% replacement of fishmeal with defatted BLM; hydrolyzed BLM, basal diet with 100% replacement of fishmeal with hydrolyzed BLM; SE, standard error; ^a,b^ means within a row with different letters are significantly different at *p* < 0.05.

**Table 4:** Effects of defatted and hydrolyzed black soldier fly larvae meal (BLM) as a fish-meal substitute on apparent total tract digestibility (ATTD) of amino acids in week 1 in weaned pigs (Exp. 1) ^1^.

Items, %	FM	Defatted BLM	Hydrolyzed BLM	SE	*p*-Value
Essential Amino Acids					
Lys	76.48	77.74	74.88	2.679	0.756
Thr	72.44	73.29	69.19	3.400	0.673
Trp	66.22	66.91	64.58	4.009	0.916
Met	79.73	75.18	72.76	3.162	0.314
Phe	76.82	75.69	74.94	2.873	0.898
Ile	62.24	63.58	60.99	4.347	0.915
Leu	70.73	69.83	68.57	3.241	0.895
His	70.82	68.55	69.51	3.058	0.871
Arg	73.35	75.31	70.53	2.942	0.528
Val	66.05	66.58	63.69	3.164	0.792
Total	71.71	71.63	69.27	3.143	0.826
Non-Essential Amino Acids					
Ala	65.73	64.08	64.41	4.108	0.956
Asp	80.57	79.49	78.30	2.471	0.813
Glu	77.65	80.17	75.96	2.730	0.561
Gly	66.60	64.47	62.99	3.789	0.798
Ser	79.03	79.37	77.97	2.547	0.922
Tyr	70.38	68.98	68.61	2.958	0.905
Cys	69.98	69.77	63.03	2.771	0.165
Pro	74.19	75.37	73.29	3.088	0.893
Total	74.70	74.69	72.52	2.923	0.833

^1^ Abbreviation: FM, basal diet with fishmeal; defatted BLM, basal diet with 100% replacement of fishmeal with defatted BLM; hydrolyzed BLM, basal diet with 100% replacement of fishmeal with hydrolyzed BLM; SE, standard error.

**Table 5:** Effects of defatted and hydrolyzed black soldier fly larvae meal (BLM) as a fish-meal substitute on apparent total tract digestibility (ATTD) of amino acids in week 2 in weaned pigs (Exp. 1) ^1^.

Items, %	FM	Defatted BLM	Hydrolyzed BLM	SE	*p*-Value
Essential Amino Acids					
Lys	79.31	79.55	74.67	1.877	0.152
Thr	74.84	76.04	69.46	2.450	0.163
Trp	70.50	71.50	68.97	2.869	0.823
Met	83.76	79.60	78.27	1.671	0.084
Phe	77.07	76.67	75.44	2.142	0.856
Ile	63.34	64.97	61.24	2.861	0.661
Leu	74.59	72.85	70.04	2.106	0.332
His	74.63	73.61	71.76	2.292	0.676
Arg	75.03	78.22	71.59	2.062	0.109
Val	67.74	68.15	63.77	2.866	0.507
Total	74.18	74.01	70.22	2.170	0.369
Non-Essential Amino Acids					
Ala	66.82	68.36	65.01	2.641	0.675
Asp	82.58	82.15	79.29	1.559	0.298
Glu	79.64	82.14	80.06	1.985	0.643
Gly	69.76	67.44	65.98	2.850	0.648
Ser	82.60	83.17	80.44	1.510	0.425
Tyr	74.02	73.08	70.71	2.307	0.591
Cys	74.77	75.65	71.10	2.071	0.287
Pro	76.74	78.38	74.13	2.028	0.354
Total	77.19	77.81	75.04	1.974	0.591

^1^ Abbreviation: FM, basal diet with fishmeal; defatted BLM, basal diet with 100% replacement of fishmeal with defatted BLM; hydrolyzed BLM, basal diet with 100% replacement of fishmeal with hydrolyzed BLM; SE, standard error.

**Table 6:** Effects of defatted and hydrolyzed black soldier fly larvae meal (BLM) as a fish-meal substitute on nitrogen (N) retention in weaned pigs (Exp. 1) ^1^.

Items	FM	Defatted BLM	Hydrolyzed BLM	SE	*p*-Value
Week 1					
N intake, g/d	18.07	16.93	18.24	1.625	0.827
N excretion in urine, g/d	2.15	2.20	2.29	0.250	0.921
N excretion in feces, g/d	4.44	3.99	4.76	0.682	0.731
Total N excretion, g/d	6.59	6.19	7.05	0.740	0.720
N retention, g/d	11.48	10.74	11.19	1.149	0.901
N retention, % of N intake	63.06	63.00	61.72	2.391	0.906
Week 2					
N intake, g/d	17.99	16.78	17.65	1.188	0.765
N excretion in urine, g/d	2.40	2.23	2.00	0.256	0.543
N excretion in feces, g/d	3.59	3.42	4.80	0.512	0.149
Total N excretion, g/d	5.99	5.65	6.80	0.404	0.155
N retention, g/d	11.99	11.13	10.85	0.881	0.644
N retention, % of N intake	66.73 ^a^	65.80 ^ab^	61.30 ^b^	1.368	0.030

^1^ Abbreviation: N, nitrogen; FM, basal diet with fishmeal; defatted BLM, basal diet with 100% replacement of fishmeal with defatted BLM; hydrolyzed BLM, basal diet with 100% replacement of fishmeal with hydrolyzed BLM; SE, standard error; ^a,b^ means within a row with different letters are significantly different at *p* < 0.05.

**Table 7:** Effects of defatted and hydrolyzed black soldier fly larvae meal (BLM) as a fish-meal substitute on blood profiles in weaned pigs (Exp. 1) ^1^.

Items	FM	Defatted BLM	Hydrolyzed BLM	SE	*p*-Value
WBCs, 10^3^/μL	21.55	21.12	21.84	0.768	0.800
RBCs, 10^6^/μL	6.54	6.64	6.89	0.248	0.601
Lymphocytes, %	59.90	61.65	60.17	2.596	0.877
Neutrophils, %	36.17	34.17	34.78	2.382	0.833
Total protein, g/dL	4.73	4.72	5.00	0.195	0.529
BUN, mg/dL	8.00 ^b^	9.33 ^ab^	10.67 ^a^	0.689	0.048

^1^ Abbreviation: WBCs, white blood cells; RBCs, red blood cells; BUN, blood urea nitrogen; FM, basal diet with fishmeal; defatted BLM, basal diet with 100% replacement of fishmeal with defatted BLM; hydrolyzed BLM, basal diet with 100% replacement of fishmeal with hydrolyzed BLM; SE, standard error; ^a,b^ means within a row with different letters are significantly different at *p* < 0.05.

**Table 8:** Effects of defatted and hydrolyzed black soldier fly larvae meal (BLM) as a fish-meal substitute on production performance in weaned pigs (Exp. 2) ^1^.

Items	FM	Defatted BLM	Hydrolyzed BLM	SE	*p*-Value
BW, kg					
Initial	8.19	8.17	8.38	0.377	0.908
Week 2	11.33	11.32	11.49	0.353	0.926
Week 4	15.88	16.87	16.41	0.422	0.276
Week 6	21.80 ^b^	23.68 ^a^	22.81 ^ab^	0.422	0.017
Weeks 0–2					
ADG, g	224.20	225.00	222.32	10.561	0.983
ADFI, g	408.33	393.30	395.49	14.365	0.730
FCR	1.82	1.75	1.78	0.112	0.721
FCG, USD/kg gain	1.26	1.14	1.18	0.074	0.566
Weeks 2–4					
ADG, g	325.27 ^b^	396.61 ^a^	351.34 ^ab^	14.975	0.010
ADFI, g	631.47	662.87	646.54	17.851	0.474
FCR	1.94	1.67	1.84	0.080	0.077
FCG, USD/kg gain	1.31 ^a^	1.10 ^b^	1.22 ^ab^	0.053	0.039
Weeks 4–6					
ADG, g	422.68	486.61	457.14	24.889	0.216
ADFI, g	962.26	991.44	976.88	19.048	0.565
FCR	2.28	2.04	2.14	0.128	0.401
FCG, USD/kg gain	1.56	1.36	1.43	0.085	0.270
Weeks 0–6					
ADG, g	324.05 ^b^	369.40 ^a^	343.60 ^ab^	7.281	0.001
ADFI, g	667.35	682.53	672.97	9.794	0.550
FCR	2.06 ^a^	1.85 ^b^	1.96 ^ab^	0.052	0.031
FCG, USD/kg gain	1.38 ^a^	1.21 ^b^	1.29 ^ab^	0.034	0.009

^1^ Abbreviation: BW, body weight, ADG, average daily gain; ADFI, average daily feed intake; FCR, feed conversion ratio; FCG, feed cost per kg gain; FM, basal diet with fishmeal; defatted BLM, basal diet with 100% replacement of fishmeal with defatted BLM; hydrolyzed BLM, basal diet with 100% replacement of fishmeal with hydrolyzed BLM; SE, standard error; ^a,b^ means within a row with different letters are significantly different at *p* < 0.05.

## Data Availability

The data presented in this study are available in this paper.
